# Supplementary figures and images for: MiRNA profiles in blood plasma from mother-child duos in human biobanks and the implication of sample quality: Circulating miRNAs as potential early markers of child health
Source: PLoS One. 2020 Apr 2;15(4):e0231040. doi: 10.1371/journal.pone.0231040 (PMC7117735; doi:10.1371/journal.pone.0231040)

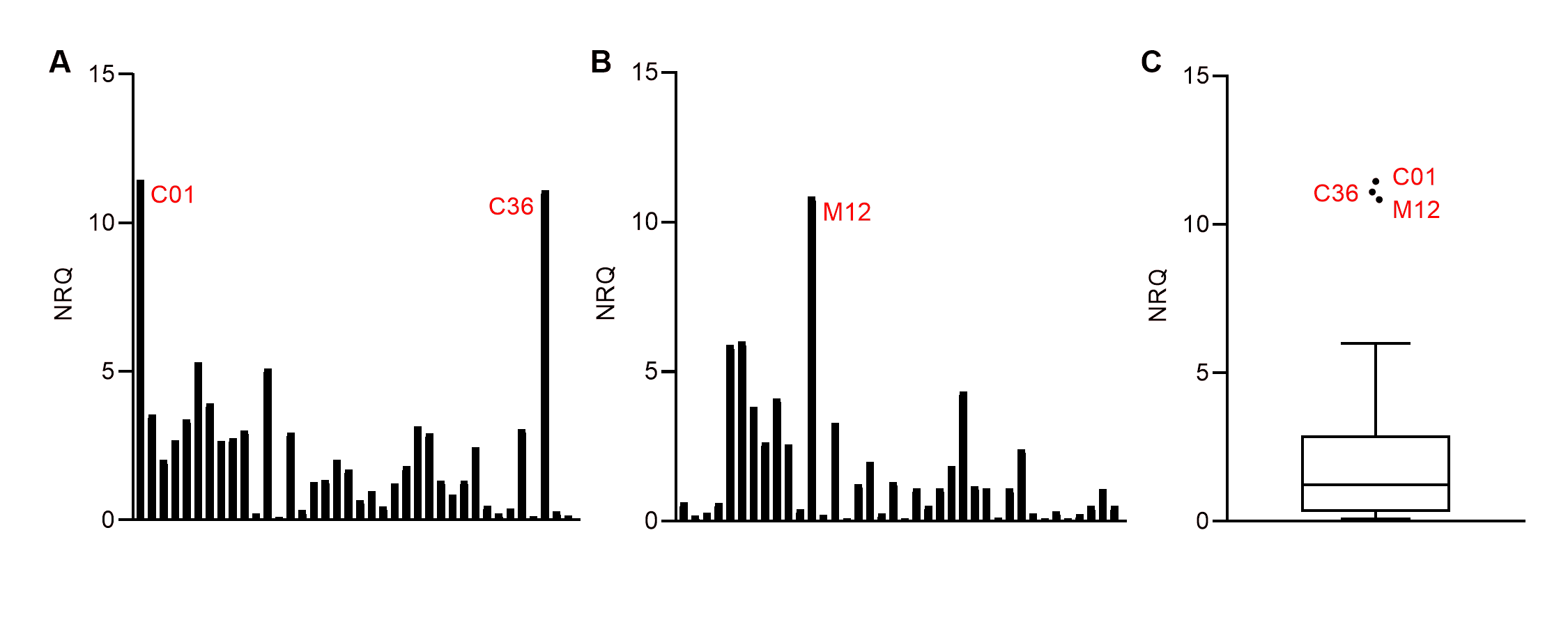

Supplement: S1 Fig — Median NRQ values per subject as bar plots for (A) cord and (B) maternal blood, and (C) all samples presented in a Tukey box and whiskers plot. (TIF) [file pone.0231040.s001.tif]
