# Supplementary material for: MiRNA profiles in blood plasma from mother-child duos in human biobanks and the implication of sample quality: Circulating miRNAs as potential early markers of child health
Source: PLoS One. 2020 Apr 2;15(4):e0231040. doi: 10.1371/journal.pone.0231040 (PMC7117735; doi:10.1371/journal.pone.0231040)
Supplement: S1 Table — Mean and standard error of the mean (SE) for raw Cq-values, Cq-values normalized with the custom spike-in, and NRQ. P-values from paired t-tests. (DOCX) [file pone.0231040.s002.docx]

**S1 Table. The 61 miRNAs of the final expression matrix.**

| **miRNA** | **Raw Cq** | | **Normalized Cq** | | **NRQ** | | **p-value** |
| --- | --- | --- | --- | --- | --- | --- | --- |
|  | **Mean ±SE** | | **Mean ±SE** | | **Mean ±SE** | |  |
|  | **Cord** | **Maternal** | **Cord** | **Maternal** | **Cord** | **Maternal** |  |
| hsa-let-7a-5p | 27.83 ±0.15 | 28.18 ±0.15 | 28.09 ±0.27 | 28.37 ±0.31 | 2.05 ±0.45 | 1.97 ±0.43 | 0.2070 |
| hsa-let-7b-5p | 29.09 ±0.23 | 29.55 ±0.14 | 29.35 ±0.27 | 29.73 ±0.31 | 1.86 ±0.27 | 1.72 ±0.32 | 0.1845 |
| hsa-let-7c-5p | 27.90 ±0.17 | 28.46 ±0.15 | 28.16 ±0.26 | 28.64 ±0.29 | 1.99 ±0.39 | 1.66 ±0.31 | 0.0526 |
| hsa-let-7d-3p | 29.87 ±0.14 | 30.74 ±0.15 | 30.13 ±0.26 | 30.93 ±0.27 | 2.15 ±0.32 | 1.38 ±0.24 | 0.0005 |
| hsa-let-7d-5p | 29.46 ±0.17 | 29.82 ±0.16 | 29.73 ±0.27 | 30.01 ±0.30 | 2.03 ±0.45 | 1.94 ±0.44 | 0.2085 |
| hsa-let-7e-5p | 28.33 ±0.16 | 28.75 ±0.13 | 28.60 ±0.28 | 28.93 ±0.29 | 2.10 ±0.41 | 1.78 ±0.35 | 0.1597 |
| hsa-let-7f-5p | 29.34 ±0.17 | 29.85 ±0.17 | 29.60 ±0.30 | 30.04 ±0.30 | 2.53 ±0.70 | 1.84 ±0.44 | 0.0736 |
| hsa-let-7g-5p | 28.93 ±0.21 | 29.60 ±0.25 | 29.19 ±0.32 | 29.79 ±0.36 | 3.23 ±1.24 | 2.87 ±1.32 | 0.0780 |
| hsa-let-7i-5p | 29.90 ±0.27 | 30.89 ±0.28 | 30.16 ±0.32 | 31.08 ±0.40 | 3.25 ±1.03 | 3.04 ±1.33 | 0.0115 |
| hsa-miR-100-5p | 30.23 ±0.38 | 31.22 ±0.19 | 30.49 ±0.39 | 31.40 ±0.32 | 13.31 ±8.84 | 1.50 ±0.27 | 0.0411 |
| hsa-miR-101-3p | 28.13 ±0.22 | 28.73 ±0.36 | 28.39 ±0.30 | 28.91 ±0.48 | 2.13 ±0.30 | 3.50 ±0.87 | 0.1972 |
| hsa-miR-103a-3p | 28.41 ±0.23 | 29.33 ±0.21 | 28.67 ±0.29 | 29.52 ±0.33 | 2.52 ±0.56 | 1.73 ±0.43 | 0.0112 |
| hsa-miR-107 | 30.79 ±0.23 | 31.69 ±0.24 | 31.05 ±0.27 | 31.87 ±0.34 | 2.27 ±0.39 | 1.87 ±0.44 | 0.0161 |
| hsa-miR-10a-5p | 30.05 ±0.30 | 30.94 ±0.14 | 30.32 ±0.33 | 31.12 ±0.28 | 4.05 ±1.66 | 1.45 ±0.30 | 0.0240 |
| hsa-miR-10b-5p | 29.20 ±0.21 | 29.95 ±0.15 | 29.47 ±0.27 | 30.13 ±0.27 | 2.27 ±0.46 | 1.43 ±0.26 | 0.0089 |
| hsa-miR-122-5p | 26.03 ±0.26 | 27.50 ±0.31 | 26.29 ±0.35 | 27.69 ±0.43 | 3.54 ±0.60 | 2.29 ±0.60 | 0.0004 |
| hsa-miR-125a-5p | 29.82 ±0.32 | 30.78 ±0.13 | 30.08 ±0.34 | 30.97 ±0.29 | 5.70 ±3.29 | 1.36 ±0.24 | 0.0209 |
| hsa-miR-125b-5p | 28.74 ±0.37 | 29.97 ±0.21 | 29.00 ±0.35 | 30.15 ±0.32 | 5.81 ±2.76 | 1.35 ±0.23 | 0.0072 |
| hsa-miR-128-3p | 31.65 ±0.20 | 32.08 ±0.22 | 31.91 ±0.28 | 32.27 ±0.35 | 2.05 ±0.40 | 2.48 ±0.71 | 0.2633 |
| hsa-miR-142-3p | 28.99 ±0.16 | 29.05 ±0.33 | 29.25 ±0.33 | 29.24 ±0.45 | 2.13 ±0.37 | 4.66 ±1.79 | 0.9658 |
| hsa-miR-148a-3p | 26.93 ±0.20 | 28.21 ±0.25 | 27.19 ±0.29 | 28.39 ±0.39 | 2.59 ±0.37 | 1.93 ±0.43 | 0.0010 |
| hsa-miR-148b-3p | 28.60 ±0.21 | 29.80 ±0.25 | 28.86 ±0.29 | 29.99 ±0.40 | 2.70 ±0.47 | 2.04 ±0.48 | 0.0028 |
| hsa-miR-151a-5p | 30.08 ±0.28 | 31.26 ±0.21 | 30.34 ±0.33 | 31.45 ±0.34 | 4.07 ±1.47 | 2.01 ±0.76 | 0.0037 |
| hsa-miR-15a-5p | 30.23 ±0.22 | 31.02 ±0.24 | 30.49 ±0.29 | 31.21 ±0.35 | 2.33 ±0.44 | 1.93 ±0.41 | 0.0261 |
| hsa-miR-15b-5p | 27.61 ±0.22 | 28.46 ±0.19 | 27.87 ±0.31 | 28.64 ±0.31 | 2.81 ±0.79 | 1.78 ±0.49 | 0.0144 |
| hsa-miR-18a-5p | 31.18 ±0.26 | 31.94 ±0.30 | 31.44 ±0.33 | 32.12 ±0.41 | 2.63 ±0.53 | 2.86 ±0.89 | 0.0845 |
| hsa-miR-191-5p | 28.74 ±0.25 | 29.23 ±0.20 | 29.00 ±0.33 | 29.42 ±0.31 | 3.03 ±0.91 | 2.81 ±1.43 | 0.1721 |
| hsa-miR-193a-5p | 30.90 ±0.22 | 31.41 ±0.12 | 31.16 ±0.31 | 31.59 ±0.28 | 2.31 ±0.44 | 1.57 ±0.32 | 0.1368 |
| hsa-miR-199a-3p | 27.30 ±0.19 | 28.38 ±0.23 | 27.56 ±0.31 | 28.56 ±0.37 | 2.69 ±0.43 | 2.01 ±0.51 | 0.0016 |
| hsa-miR-19a-3p | 26.76 ±0.26 | 27.57 ±0.33 | 27.02 ±0.33 | 27.76 ±0.45 | 2.75 ±0.55 | 3.62 ±1.36 | 0.0862 |
| hsa-miR-19b-3p | 26.78 ±0.25 | 27.73 ±0.32 | 27.04 ±0.31 | 27.91 ±0.44 | 2.66 ±0.51 | 3.36 ±1.30 | 0.0330 |
| hsa-miR-21-5p | 24.44 ±0.24 | 25.14 ±0.17 | 24.70 ±0.33 | 25.33 ±0.32 | 2.83 ±0.64 | 1.77 ±0.37 | 0.0653 |
| hsa-miR-221-3p | 29.27 ±0.28 | 30.14 ±0.18 | 29.53 ±0.35 | 30.32 ±0.31 | 3.80 ±1.04 | 1.75 ±0.46 | 0.0245 |
| hsa-miR-222-3p | 27.88 ±0.28 | 28.74 ±0.17 | 28.14 ±0.32 | 28.92 ±0.28 | 2.95 ±0.71 | 1.50 ±0.35 | 0.0220 |
| hsa-miR-22-3p | 28.46 ±0.30 | 29.03 ±0.26 | 28.72 ±0.35 | 29.22 ±0.37 | 2.78 ±0.67 | 2.32 ±0.51 | 0.1796 |
| hsa-miR-22-5p | 31.59 ±0.28 | 31.91 ±0.19 | 31.85 ±0.33 | 32.10 ±0.31 | 2.70 ±0.89 | 1.90 ±0.37 | 0.4613 |
| hsa-miR-23a-3p | 25.00 ±0.26 | 25.48 ±0.13 | 25.26 ±0.33 | 25.66 ±0.28 | 2.78 ±0.70 | 1.62 ±0.32 | 0.2112 |
| hsa-miR-23b-3p | 25.46 ±0.26 | 25.90 ±0.14 | 25.72 ±0.32 | 26.09 ±0.28 | 2.61 ±0.67 | 1.64 ±0.32 | 0.2129 |
| hsa-miR-24-3p | 27.41 ±0.34 | 28.03 ±0.20 | 27.67 ±0.35 | 28.22 ±0.32 | 4.45 ±2.34 | 1.94 ±0.46 | 0.1534 |
| hsa-miR-25-3p | 25.44 ±0.20 | 26.38 ±0.18 | 25.70 ±0.26 | 26.57 ±0.31 | 2.16 ±0.29 | 1.46 ±0.28 | 0.0069 |
| hsa-miR-26b-5p | 27.62 ±0.24 | 28.41 ±0.17 | 27.89 ±0.31 | 28.60 ±0.30 | 2.95 ±0.78 | 1.67 ±0.41 | 0.0278 |
| hsa-miR-29a-3p | 29.16 ±0.32 | 29.37 ±0.20 | 29.42 ±0.40 | 29.55 ±0.30 | 3.36 ±1.18 | 1.96 ±0.43 | 0.7245 |
| hsa-miR-29c-3p | 28.75 ±0.31 | 29.05 ±0.21 | 29.01 ±0.39 | 29.24 ±0.30 | 3.25 ±1.08 | 1.87 ±0.38 | 0.5511 |
| hsa-miR-30c-5p | 28.22 ±0.20 | 28.96 ±0.17 | 28.48 ±0.28 | 29.14 ±0.29 | 2.28 ±0.43 | 1.53 ±0.28 | 0.0185 |
| hsa-miR-30e-5p | 26.88 ±0.30 | 27.97 ±0.20 | 27.14 ±0.33 | 28.16 ±0.30 | 3.97 ±1.58 | 1.38 ±0.24 | 0.0080 |
| hsa-miR-335-5p | 30.76 ±0.27 | 32.10 ±0.26 | 31.02 ±0.33 | 32.29 ±0.36 | 4.07 ±1.29 | 2.06 ±0.74 | 0.0013 |
| hsa-miR-34a-5p | 32.80 ±0.25 | 33.50 ±0.17 | 33.06 ±0.27 | 33.69 ±0.25 | 2.28 ±0.58 | 1.33 ±0.21 | 0.0433 |
| hsa-miR-34b-5p | 29.61 ±0.19 | 29.69 ±0.16 | 29.87 ±0.22 | 29.88 ±0.23 | 1.44 ±0.19 | 1.62 ±0.30 | 0.9471 |
| hsa-miR-365a-3p | 29.36 ±0.33 | 30.50 ±0.18 | 29.62 ±0.36 | 30.69 ±0.32 | 5.60 ±2.98 | 1.42 ±0.25 | 0.0118 |
| hsa-miR-373-5p | 26.65 ±0.27 | 27.67 ±0.22 | 26.91 ±0.36 | 27.86 ±0.33 | 4.91 ±1.91 | 1.94 ±0.67 | 0.0007 |
| hsa-miR-423-3p | 30.26 ±0.28 | 31.70 ±0.27 | 30.53 ±0.30 | 31.89 ±0.38 | 3.15 ±0.59 | 1.76 ±0.40 | 0.0006 |
| hsa-miR-423-5p | 28.18 ±0.23 | 29.36 ±0.19 | 28.44 ±0.30 | 29.55 ±0.32 | 3.10 ±0.69 | 1.67 ±0.45 | 0.0007 |
| hsa-miR-424-5p | 26.24 ±0.31 | 27.55 ±0.29 | 26.50 ±0.34 | 27.73 ±0.42 | 4.85 ±2.21 | 1.92 ±0.37 | 0.0079 |
| hsa-miR-425-5p | 29.96 ±0.25 | 30.87 ±0.22 | 30.22 ±0.31 | 31.05 ±0.33 | 2.93 ±0.81 | 2.03 ±0.76 | 0.0104 |
| hsa-miR-484 | 29.03 ±0.21 | 29.87 ±0.19 | 29.29 ±0.24 | 30.05 ±0.27 | 1.98 ±0.26 | 1.50 ±0.33 | 0.0023 |
| hsa-miR-497-5p | 30.88 ±0.20 | 31.59 ±0.26 | 31.14 ±0.29 | 31.78 ±0.36 | 2.07 ±0.28 | 1.91 ±0.33 | 0.0187 |
| hsa-miR-517a-3p | 30.25 ±0.50 | 31.15 ±0.28 | 30.51 ±0.52 | 31.34 ±0.37 | 74.51 ±59.24 | 1.94 ±0.48 | 0.1380 |
| hsa-miR-652-3p | 30.24 ±0.30 | 31.61 ±0.22 | 30.50 ±0.35 | 31.80 ±0.36 | 4.70 ±1.59 | 1.73 ±0.50 | 0.0017 |
| hsa-miR-660-5p | 30.27 ±0.24 | 31.33 ±0.25 | 30.53 ±0.30 | 31.51 ±0.36 | 2.67 ±0.50 | 1.80 ±0.38 | 0.0109 |
| hsa-miR-93-5p | 27.66 ±0.25 | 28.58 ±0.28 | 27.92 ±0.29 | 28.77 ±0.40 | 2.37 ±0.40 | 2.44 ±0.66 | 0.0326 |
| hsa-miR-99a-5p | 29.33 ±0.35 | 30.38 ±0.21 | 29.59 ±0.36 | 30.57 ±0.32 | 6.43 ±3.34 | 1.59 ±0.34 | 0.0200 |

Mean and standard error of the mean (SE) for raw *Cq*-values, *Cq*-values normalized with the custom spike-in, and NRQ. P-values from paired t-tests.
