# Supplementary material for: MiRNA profiles in blood plasma from mother-child duos in human biobanks and the implication of sample quality: Circulating miRNAs as potential early markers of child health
Source: PLoS One. 2020 Apr 2;15(4):e0231040. doi: 10.1371/journal.pone.0231040 (PMC7117735; doi:10.1371/journal.pone.0231040)
Supplement: S2 Table — (DOCX) [file pone.0231040.s003.docx]

**S2 Table. R^2^ and p-values from linear regression analysis for each miRNA.**

| **miRNA** | **R^2^** | **p-value** |
| --- | --- | --- |
| hsa-miR-142-3p | 0,6374 | <0,0001 |
| hsa-miR-34b-5p | 0,6256 | <0,0001 |
| hsa-let-7a-5p | 0,5370 | <0,0001 |
| hsa-miR-373-5p | 0,5248 | <0,0001 |
| hsa-let-7d-5p | 0,5021 | <0,0001 |
| hsa-miR-497-5p | 0,4918 | <0,0001 |
| hsa-let-7d-3p | 0,4813 | <0,0001 |
| hsa-let-7f-5p | 0,4750 | <0,0001 |
| hsa-let-7e-5p | 0,4493 | <0,0001 |
| hsa-miR-199a-3p | 0,3990 | <0,0001 |
| hsa-let-7c-5p | 0,3826 | <0,0001 |
| hsa-miR-10b-5p | 0,3643 | <0,0001 |
| hsa-miR-122-5p | 0,3594 | <0,0001 |
| hsa-miR-484 | 0,3565 | <0,0001 |
| hsa-miR-101-3p | 0,3240 | 0,0002 |
| hsa-miR-191-5p | 0,3167 | 0,0002 |
| hsa-miR-30c-5p | 0,3135 | 0,0003 |
| hsa-miR-193a-5p | 0,3045 | 0,0003 |
| hsa-let-7i-5p | 0,3039 | 0,0003 |
| hsa-miR-15a-5p | 0,3010 | 0,0004 |
| hsa-miR-23b-3p | 0,3001 | 0,0004 |
| hsa-miR-423-5p | 0,2938 | 0,0004 |
| hsa-miR-425-5p | 0,2886 | 0,0005 |
| hsa-miR-148a-3p | 0,2873 | 0,0005 |
| hsa-let-7b-5p | 0,2867 | 0,0005 |
| hsa-miR-15b-5p | 0,2825 | 0,0006 |
| hsa-miR-148b-3p | 0,2710 | 0,0008 |
| hsa-let-7g-5p | 0,2694 | 0,0008 |
| hsa-miR-128-3p | 0,2624 | 0,0010 |
| hsa-miR-22-3p | 0,2509 | 0,0014 |
| hsa-miR-21-5p | 0,2476 | 0,0015 |
| hsa-miR-19b-3p | 0,2439 | 0,0016 |
| hsa-miR-26b-5p | 0,2357 | 0,0020 |
| hsa-miR-18a-5p | 0,2353 | 0,0020 |
| hsa-miR-221-3p | 0,2257 | 0,0026 |
| hsa-miR-23a-3p | 0,2209 | 0,0029 |
| hsa-miR-19a-3p | 0,2196 | 0,0030 |
| hsa-miR-107 | 0,2129 | 0,0035 |
| hsa-miR-103a-3p | 0,2113 | 0,0037 |
| hsa-miR-29a-3p | 0,2080 | 0,0040 |
| hsa-miR-423-3p | 0,2073 | 0,0041 |
| hsa-miR-335-5p | 0,2025 | 0,0046 |
| hsa-miR-22-5p | 0,1999 | 0,0049 |
| hsa-miR-25-3p | 0,1979 | 0,0051 |
| hsa-miR-151a-5p | 0,1959 | 0,0054 |
| hsa-miR-93-5p | 0,1873 | 0,0066 |
| hsa-miR-29c-3p | 0,1768 | 0,0086 |
| hsa-miR-652-3p | 0,1715 | 0,0098 |
| hsa-miR-222-3p | 0,1697 | 0,0102 |
| hsa-miR-660-5p | 0,1619 | 0,0123 |
| hsa-miR-24-3p | 0,1405 | 0,0204 |
| hsa-miR-10a-5p | 0,1367 | 0,0224 |
| hsa-miR-30e-5p | 0,1244 | 0,0298 |
| hsa-miR-424-5p | 0,1180 | 0,0347 |
| hsa-miR-34a-5p | 0,1174 | 0,0352 |
| hsa-miR-125a-5p | 0,1005 | 0,0524 |
| hsa-miR-365a-3p | 0,0994 | 0,0539 |
| hsa-miR-99a-5p | 0,0959 | 0,0586 |
| hsa-miR-125b-5p | 0,0720 | 0,1033 |
| hsa-miR-100-5p | 0,0718 | 0,1038 |
| hsa-miR-517a-3p | 0,0713 | 0,1052 |
