# Supplementary material for: MiRNA profiles in blood plasma from mother-child duos in human biobanks and the implication of sample quality: Circulating miRNAs as potential early markers of child health
Source: PLoS One. 2020 Apr 2;15(4):e0231040. doi: 10.1371/journal.pone.0231040 (PMC7117735; doi:10.1371/journal.pone.0231040)
Supplement: S3 Table — WB: Whole blood; WBCs: White blood cells; PBMCs: Peripheral blood mononuclear cells. (DOCX) [file pone.0231040.s004.docx]

**S3 Table. MiRNAs whose expression levels have been implicated in ADHD in previous research.**

| **miRNA** | **Status in ADHD** | **Sample type** | **References** |
| --- | --- | --- | --- |
| hsa-let-7d | Increased | Serum | (1) |
| hsa-let-7d | Increased | Serum | (2) |
| hsa-let-7d | Decreased | WB | (3) |
| hsa-let-7g-5p | ADHD-prediction model | WBCs | (4) |
| hsa-miR-101-3p | ADHD-prediction model | WBCs | (4) |
| hsa-miR-101-3p | Increased | Serum | (5) |
| hsa-miR-106b-5p | Decreased | Serum | (5) |
| hsa-miR-106b-5p | Decreased | WB | (6) |
| hsa-miR-107 | Decreased | WB | (6) |
| hsa-miR-126-5p | ADHD-prediction model | WBCs | (4) |
| hsa-miR-126-5p | Increased | WBCs | (7) |
| hsa-miR-130a-3p | Increased | Serum | (5) |
| hsa-miR-138-5p | Increased | Serum | (5) |
| hsa-miR-140-3p | ADHD-prediction model | WBCs | (4) |
| hsa-miR-140-3p | Increased | WBCs | (7) |
| hsa-miR-142-3p | Decreased levels in ADHD group when presence of psychiatric disease in their families. | Plasma | (8) |
| hsa-miR-142-5p | ADHD-prediction model | WBCs | (4) |
| hsa-miR-150-5p | ADHD-prediction model | WBCs | (4) |
| hsa-miR-151a-3p | ADHD-prediction model | WBCs | (4) |
| hsa-miR-151a-5p | ADHD-prediction model | WBCs | (4) |
| hsa-miR-155a-5p | Increased | WB | (6) |
| hsa-miR-185b-5p | Decreased | PBMCs | (9) |
| hsa-miR-18a-5p | Decreased | WB | (6) |
| hsa-miR-191-5p | Increased | PBMCs | (9) |
| hsa-miR-195-5p | Increased | Serum | (5) |
| hsa-miR-22-3p | Decreased | WB | (6) |
| hsa-miR-223-3p | ADHD-prediction model | WBCs | (4) |
| hsa-miR-24-3p | Decreased | WB | (6) |
| hsa-miR-26b-5p | Decreased | PBMCs | (9) |
| hsa-miR-27a-3p | ADHD-prediction model | WBCs | (4) |
| hsa-miR-30e-5p | ADHD-prediction model | WBCs | (4) |
| hsa-miR-30e-5p | Increased | WBCs | (7) |
| hsa-miR-34c-3p | Increased | PBMCs | (10) |
| hsa-miR-378 | Decreased levels in ADHD group when presence of psychiatric disease in their families. | Plasma | (8) |
| hsa-miR-486-5p | ADHD-prediction model | WBCs | (4) |
| hsa-miR-5692b | Increased | WB | (3) |
| hsa-miR-92a-3p | ADHD-prediction model | WBCs | (4) |

WB: Whole blood; WBCs: White blood cells; PBMCs: Peripheral blood mononuclear cells.

**References**

1. Cao P, Wang L, Cheng Q, Sun X, Kang Q, Dai L, et al. Changes in serum miRNA-let-7 level in children with attention deficit hyperactivity disorder treated by repetitive transcranial magnetic stimulation or atomoxetine: An exploratory trial. Psychiatry research. 2019;274:189-94.

2. Wu LH, Peng M, Yu M, Zhao QL, Li C, Jin YT, et al. Circulating MicroRNA Let-7d in Attention-Deficit/Hyperactivity Disorder. Neuromolecular medicine. 2015;17(2):137-46.

3. Aydin SU, Kabukcu Basay B, Cetin GO, Gungor Aydin A, Tepeli E. Altered microRNA 5692b and microRNA let-7d expression levels in children and adolescents with attention deficit hyperactivity disorder. Journal of psychiatric research. 2019;115:158-64.

4. Wang LJ, Li SC, Lee MJ, Chou MC, Chou WJ, Lee SY, et al. Blood-Bourne MicroRNA Biomarker Evaluation in Attention-Deficit/Hyperactivity Disorder of Han Chinese Individuals: An Exploratory Study. Frontiers in psychiatry. 2018;9:227.

5. Zadehbagheri F, Hosseini E, Bagheri-Hosseinabadi Z, Rekabdarkolaee HM, Sadeghi I. Profiling of miRNAs in serum of children with attention-deficit hyperactivity disorder shows significant alterations. Journal of psychiatric research. 2019;109:185-92.

6. Kandemir H, Erdal ME, Selek S, Ay OI, Karababa IF, Kandemir SB, et al. Evaluation of several micro RNA (miRNA) levels in children and adolescents with attention deficit hyperactivity disorder. Neuroscience letters. 2014;580:158-62.

7. Wang LJ, Li SC, Kuo HC, Chou WJ, Lee MJ, Chou MC, et al. Gray matter volume and microRNA levels in patients with attention-deficit/hyperactivity disorder. European archives of psychiatry and clinical neuroscience. 2019.

8. Karadag M, Gokcen C, Nacarkahya G, Namiduru D, Dandil F, Calisgan B, et al. Chronotypical characteristics and related miR-142-3p levels of children with attention deficit and hyperactivity disorder. Psychiatry research. 2018;273:235-9.

9. Sanchez-Mora C, Soler Artigas M, Garcia-Martinez I, Pagerols M, Rovira P, Richarte V, et al. Epigenetic signature for attention-deficit/hyperactivity disorder: identification of miR-26b-5p, miR-185-5p, and miR-191-5p as potential biomarkers in peripheral blood mononuclear cells. Neuropsychopharmacology : official publication of the American College of Neuropsychopharmacology. 2018.

10. Garcia-Martinez I, Sanchez-Mora C, Pagerols M, Richarte V, Corrales M, Fadeuilhe C, et al. Preliminary evidence for association of genetic variants in pri-miR-34b/c and abnormal miR-34c expression with attention deficit and hyperactivity disorder. Translational psychiatry. 2016;6(8):e879.
