# Supplementary material for: MiRNA profiles in blood plasma from mother-child duos in human biobanks and the implication of sample quality: Circulating miRNAs as potential early markers of child health
Source: PLoS One. 2020 Apr 2;15(4):e0231040. doi: 10.1371/journal.pone.0231040 (PMC7117735; doi:10.1371/journal.pone.0231040)
Supplement: Attachment — Submitted filename: response_to_reviewers.docx [file pone.0231040.s005.docx]

Zhong-Cheng Luo

Academic Editor

PLOS ONE

Oslo, 06.03.2020

**Dear Dr. Luo**

Subject: Submission of revised manuscript PONE-D-19-35144

Thank you for the opportunity to submit a revised version of our manuscript “MiRNA profiles in blood plasma from mother-child duos in human biobanks and the implication of sample quality: Circulating miRNAs as potential early markers of child health”. We appreciate the time and effort that you and the reviewers have dedicated to provide valuable feedback, and we believe the comments raised by the reviewers and our resulting revision has improved our manuscript.

I have included the reviewers’ comments and our responses below.

We hope the revised version is now suitable for publication in PLOS ONE and look forward to hearing from you.

Kind regards,

Lene Brattsti Dypås, MSc

PhD Candidate

*Department of Environmental Health*

*Norwegian Institute of Public Health,*

*P.O. Box 222 Skøyen,*

*N-0213 OSLO, Norway*

*Telephone +47 48 12 66 98*

Email: lenebrattsti.dypas@fhi.no

**Reviewer comments, author responses and manuscript changes**

**Reviewer #1**

*Comment 1: Although the authors mention performing correlation analysis between each miRNA for the 38 pairs, these data are not included in manuscript. As the data in Figure 4 suggests that there may actually a large group of miRNAs with higher expression levels (>log2(0)) that correlate between cord and maternal samples, it would be important to include the R2 and p-values for each miRNA correlation as a supplement in the manuscript. P-value for the linear regression analysis in Figure 4 should also be included.*

Response: We agree that this is valuable information and Figure 4 has been revised to include the p-value for the linear regression analysis, and, a new table (S2 table) containing the R^2^ and p-values for each miRNA correlation has been included in supplementary information.

*Comment 2: In methods, the authors state that they collected 50 pairs of cord blood/maternal blood. However, there are only 38 pairs included in the miRNA analysis, with no explanation for the exclusion of other sample pairs. Please include a statement on why 12 pairs were excluded from miRNA analysis, as this could be an important technical caveat.*

Response: Thank you for pointing out that this important piece of information was missing. The manuscript has been revised to include a statement explaining that 12 pairs were excluded due to insufficient sample volume (line 111-112).

*Comment 3: No statement of patient consent prior to sample collection. This should be included.*

Response: A statement of patient consent prior to sample collection has been included in the methods section (line 117-118).

*Comment 4: Figure 3: Individual points for each sample should be shown in the box plots.*

Response: Figure 3 has been revised to include individual points for each sample as we agree that this enhances the readability of the plot and adds value.

*Comment 5: How might altered levels of exosomes affect miRNA analysis? Is there a difference in exosomes between cord blood and maternal blood?*

Response: The questions raised here is a very interesting topic, with no clear answers as of yet. The manuscript has been revised to include a paragraph on miRNAs in exosomes to clarify (line 224-229).

Early research hypothesized that extracellular miRNAs were protected by encapsulation in exosomes, and that the majority of miRNAs found in plasma or serum stemmed from these vesicles (1). Later it was discovered that 90-99% of extracellular miRNAs were actually associated with proteins in the argonaute family, not exosomes (2, 3). Moreover, it has also been shown that exosomes might actually contain very small amounts of miRNA, contrary to what was hypothesized before (4), and therefore might not affect analysis of total circulating miRNAs to such an extent as previously believed. Either way, when analyzing circulating miRNAs there is no precedence to remove exosomes from plasma, and miRNAs in exosomes are regarded as part of the circulating (i.e. extracellular) miRNAs, and altered levels of exosomes containing miRNAs then reflect changes in the individual’s physical state causing altered levels of circulating miRNAs. However, it is important to always include details of plasma preparation steps in your protocol so that readers know if exosomes are included in the plasma (or other sample material) for reproducibility and comparative purposes.

Regarding your question on a difference in exosomes between cord and maternal blood, not much is known. There is however one small study on this in serum, where they found slightly higher concentrations of exosomes in serum from maternal blood compared to cord blood (5).

**Reviewer #2**

*Comment 1: Line 180 : NRQ = 2-ΔCq (where ΔCq = ΔCq (target) - Cq (global mean)). Please confirm ΔCq (target) is used but not Cq (target); also “global mean” is not defined.*

Response: Thank you for pointing this out. Δ*Cq*(target) is a typo, and should be *Cq*(target) as you correctly assumed. The typo has been corrected and a definition of “global mean” has been added (line 183-184).

**References**

1.	Hunter MP, Ismail N, Zhang X, Aguda BD, Lee EJ, Yu L, et al. Detection of microRNA expression in human peripheral blood microvesicles. PLoS One. 2008;3(11):e3694-e.

2.	Arroyo JD, Chevillet JR, Kroh EM, Ruf IK, Pritchard CC, Gibson DF, et al. Argonaute2 complexes carry a population of circulating microRNAs independent of vesicles in human plasma. Proc Natl Acad Sci U S A. 2011;108(12):5003-8.

3.	Turchinovich A, Weiz L, Langheinz A, Burwinkel B. Characterization of extracellular circulating microRNA. Nucleic Acids Res. 2011;39(16):7223-33.

4.	Chevillet JR, Kang Q, Ruf IK, Briggs HA, Vojtech LN, Hughes SM, et al. Quantitative and stoichiometric analysis of the microRNA content of exosomes. Proc Natl Acad Sci U S A. 2014;111(41):14888-93.

5.	Jia L, Zhou X, Huang X, Xu X, Jia Y, Wu Y, et al. Maternal and umbilical cord serum-derived exosomes enhance endothelial cell proliferation and migration. FASEB J. 2018;32(8):4534-43.
